# Prognostic Value of Isolated Sarcopenia or Malnutrition–Sarcopenia Syndrome for Clinical Outcomes in Hospitalized Patients

**DOI:** 10.3390/nu14112207

**Published:** 2022-05-26

**Authors:** Iasmin Matias Sousa, Camila Ferri Burgel, Flávia Moraes Silva, Ana Paula Trussardi Fayh

**Affiliations:** 1 Postgraduate Program in Health Sciences, Health Sciences Center, Federal University of Rio Grande do Norte, Natal 59012-570, Brazil; iasmin_matias@hotmail.com; 2 Nutrition and Dietetic Service, Complex Hospital of Santa Casa de Misericórdia de Porto Alegre, Porto Alegre 90020-090, Brazil; camilaburgel@hotmail.com; 3 Department of Nutrition, Federal University of Health Sciences of Porto Alegre, Porto Alegre 90050-170, Brazil; flavia.moraes.silva@hotmail.com; 4 Postgraduate Program in Nutrition and Health Sciences, Health Sciences Center, Federal University of Rio Grande do Norte, Natal 59078-790, Brazil

**Keywords:** sarcopenia, malnutrition, length of stay, mortality, hospital readmission

## Abstract

Malnutrition–sarcopenia syndrome (MSS) is frequent in the hospital setting. However, data on the predictive validity of sarcopenia and MSS are scarce. We evaluated the association between sarcopenia and MSS and clinical adverse outcomes (prolonged length of hospital stay—LOS, six-month readmission, and death) using a prospective cohort study involving adult hospitalized patients (*n* = 550, 55.3 ± 14.9 years, 53.1% males). Sarcopenia was diagnosed according to the EWGSOP2, and malnutrition according to the Subjective Global Assessment (SGA). Around 34% were malnourished, 7% probable sarcopenic, 15% sarcopenic, and 2.5% severe sarcopenic. In-hospital death occurred in 12 patients, and the median LOS was 10.0 days. Within six months from discharge, 7.9% of patients died, and 33.8% were readmitted to the hospital. Probable sarcopenia/sarcopenia had increased 3.95 times (95% CI 1.11–13.91) the risk of in-hospital death and in 3.25 times (95% CI 1.56–6.62) the chance of mortality in six months. MSS had increased the odds of prolonged LOS (OR = 2.73; 95% CI 1.42–5.25), readmission (OR = 7.64; 95% CI 3.06–19.06), and death (OR = 1.15; 95% CI 1.08–1.21) within six months after discharge. Sarcopenia and MSS were predictors of worse clinical outcomes in hospitalized patients.

## 1. Introduction

The profile of hospitalized patients is marked by varying degrees of nutritional depletion, represented by conditions such as malnutrition and sarcopenia [1]. Sarcopenia is a progressive and generalized muscle disease associated with adverse outcomes in different clinical scenarios [2]. Malnutrition is a combination of compromised food intake, or assimilation of nutrients, with or without different degrees of inflammation, having as consequence changes on body composition and biological function [3,4].

The severity and prevalence of sarcopenia significantly increase with age, varying from 9.9% to 40.4% in community-dwelling adults, 28% to 69% in hospitalized patients, and 71% in those in intensive care units, depending on the diagnostic tool used [5,6,7,8]. It is widely described in the literature that malnutrition is highly prevalent and a strong predictor of worse clinical outcomes in hospitalized patients [9]. Sarcopenia is also a condition recognized for increasing the risk of worse clinical outcomes, with a large literature available, especially in non-hospitalized individuals (with impairment of daily activities and instrumental activities of daily living, risk of hip fracture, and mortality) [10,11,12]. Some studies conducted in hospitalized patients have demonstrated poorer mobility, low quality of life, increased length of hospital stay, post-surgical complications, and mortality in patients with sarcopenia, compared to those without it [1,12,13,14,15].

In hospitalized patients, the risk of sarcopenia and other syndromes, such as cachexia and malnutrition, is significantly high due to acute diseases, mobility impairment, and anorexia [16]. Even though they are defined as different conditions, there is an overlap between sarcopenia and malnutrition, as they have similar etiological factors and the loss of body tissue as a common feature. As a result, there is a complex relationship between them, and each condition is increased by the other [17,18]. The co-existence of sarcopenia and malnutrition in hospitalized patients is frequent. A systematic review of seven studies (2506 patients) revealed a high association [OR: 4.06 (95% CI: 2.43, 6.80), I^2^ = 71.4%] and considerable overlap (41.6%) between sarcopenia and (risk of) malnutrition in older hospitalized adults [19]. In addition, when these conditions co-exist, also named malnutrition–sarcopenia syndrome (MSS), they can be a predictor of mortality in older hospitalized patients and a prognostic factor in their management [20,21]. The scarce literature on the prognostic value of this syndrome, not considering elderly patients, and clinical outcomes different from mortality, provides a gap for new studies in the area.

We have hypothesized that patients with sarcopenia present longer hospital stay and a higher risk of in-hospital death, and worse outcomes after discharge in comparison to patients without sarcopenia. In addition, if patients present MSS, their prognostic is worse. Therefore, this study aimed to evaluate sarcopenia isolated, or co-existing with malnutrition, as a predictor of clinical outcomes (prolonged length of hospital stay, hospital readmission, and death) in hospitalized patients.

## 2. Materials and Methods

### 2.1. Design and Subjects

This is a secondary analysis of a prospective cohort study involving hospitalized patients [22]. The study was conducted between September 2018 and February 2020, including adult inpatients (≥18 years) from both sexes, from five hospitals, in a hospital complex in Porto Alegre (Brazil). The sample was selected among all patients admitted to the hospital (except emergency and intensive care units—ICU) who met the inclusion criteria. Patients with no possibility to communicate or walk, unable to measure handgrip strength (HGS), presenting leg edema, pregnant or lactating, were not included. The Hospital Ethics Committee approved the protocol (under number 2.735.945), and all participants signed a term of informed consent.

### 2.2. Procedures

Clinical and sociodemographic data were obtained from the digital records at the hospital, including age, sex, self-reported ethnicity, reason for hospitalization (date of admission and discharge), and comorbidities (for example, hypertension, diabetes, cardiovascular disease). The Charlson comorbidity index (CCI), adjusted for age, was calculated to evaluate the severity of the disease in each patient by using an electronic application recommended by Hall et al. [23,24]. Trained registered dietitians performed the nutritional assessment within the first 48 h after hospital admission.

### 2.3. Nutrition Evaluation

Bodyweight and height were measured using a Filizola^®^ scale with an attached stadiometer. Calf circumference (CC) was measured with an inelastic tape (Sanny), with individuals seated with their legs positioned at a 90° angle. We classified low muscle mass according to the cutoff points validated for the Brazilian population (≤34 cm for males and ≤33 cm for females) [25].

The subjective global assessment (SGA) was adopted for the diagnosis of malnutrition. We conducted a face-to-face interview, including questions about bodyweight history, food intake, gastrointestinal symptoms, and functional capacity, combined with a physical examination to identify fat and muscle mass loss and fluid accumulation, and also the determination of the metabolic demand of the disease. Considering all these features, patients were subjectively classified as well-nourished (SGA A), moderately (or suspected of being) malnourished (SGA B), or severely malnourished (SGA C) [26]. For data analyses, we grouped SGA B and C and named it malnutrition.

### 2.4. Muscle Strength and Physical Performance Assessment

Handgrip strength (HGS) was measured using a hydraulic hand dynamometer (Saehan^®^); this process included familiarization with the device and triplicate measurements, with maximum strength for three seconds using the non-dominant hand. For HGS, patients were instructed to be seated with elbows flexed at 90° at the side of the body. The highest recorded value was used as maximum muscular strength. Low HGS was classified according to the cutoff point proposed by the EWGSOP2 [2]: <27 kg for males and <16 kg for females.

The physical performance was accessed by the “timed up-and-go” (TUG) test. For it, patients were asked to get up from a chair, walk to a marker three meters away, turn 180°, walk back, and sit down again. The researcher timed each patient, and physical performance was defined as poor when the result of the TUG was >20 s [2].

### 2.5. Definition of Sarcopenia

Individuals were classified according to the EWGSOP2 [2]: (1) those with low muscle strength (only) were named “probable sarcopenic”, (2) those with low muscle strength + low muscle mass (low CC) were named “sarcopenic”, and (3) those with low muscle strength + low muscle mass + low physical performance were named “severe sarcopenic”. For data analysis, we grouped probable sarcopenic and sarcopenic patients.

### 2.6. Outcomes

We collected the outcomes of length-of-hospital stay (LOS) and in-hospital death from the electronic medical records. The outcomes for hospital readmission and death were collected by phone call within six months after discharge.

### 2.7. Statistical Analysis

The calculation of the sample size considered the difference in the mortality incidence between patients with and without sarcopenia (27% versus 10%), observed in the study by Gariballa et al. [27], a power of 80%, a significance level of 5%, and an additional of 20% for adjustment in multivariate analysis, resulting in a sample of 240 patients. The calculation was performed on the online calculator OpenEpi (https://www.openepi.com/Menu/OE_Menu.htm, accessed 10 October 2021).

Descriptive statistics were also calculated. Mean and standard deviation for parametric quantitative variables, median and interquartile range for non-parametric variables, and absolute and relative frequency for categorical variables. Kolmogorov–Smirnov was used for testing the quantitative variable normality.

For the data analysis, we grouped patients with probable sarcopenia and those with sarcopenia, comparing them to non-sarcopenic ones by Student’s *t*-test, Mann–Whitney test, chi-squared, or Fisher’s exact test in bivariate analysis. Multivariate analyses were performed considering the outcomes in-hospital death (Cox regression), prolonged LOS (categorized by a median of 10 days considering its data distribution in our sample), death six months after discharge, and hospital readmission six months after discharge (logistic regression). Variables included in multivariate analysis were selected by the *p* value (*p* < 0.20) obtained on bivariate analysis for comparison of patients with and without sarcopenia; thus, the CCI, adjusted for age and surgical procedure, was considered as a confounder in the analyses. BMI was not included in the models due to its strong relation with malnutrition, while age is one component of adjusted-age CCI. Additionally, CC and HGS are criteria for sarcopenia diagnosis and it precludes their inclusion in the models constructed. In the multivariate analysis, we constructed independent models, considering sarcopenia, malnutrition, and MSS as independent predictors of outcomes.

The entire analysis was performed in SPPS 22.0 software, and *p* values < 0.05 were considered statistically significant.

## 3. Results

### 3.1. General Features of Patients

A total of 550 patients were included in the current analysis since the primary cohort study included 600 patients and the CC data from 50 patients could not be obtained due to leg edema. The mean age of patients was equal to 55.3 ± 14.9 years; 77.3% (*n* = 425) self-reported white ethnicity, and 53.1% (*n* = 292) were males.

More than half of the patients (*n* = 285; 51.8%) were admitted to the hospital due to cancer diagnosis, 13.1% (*n* = 72) due to cardiac diseases, 10.5% due to lung diseases (*n* = 58), and 8.7% (*n* = 48) due to gastrointestinal diseases. The median of CCI adjusted for age was equal to 4.0 (2.0–6.0) points.

The mean of actual body weight and BMI was 73.7 ± 17.0 kg and 27.5 ± 5.5 kg/m^2^, respectively. The mean of HGS was 34.3 ± 9.6 for males and 20.8 ± 5.7 for females, while the mean of CC was 36.9 ± 4.1 for males and 36.1 ± 4.5 for females; 17.3% of the sample (*n* = 95) presented low HGS and 24.7% (*n* = 136) presented low CC. The majority of patients (*n* = 357; 65.9%) presented reduced functional capacity. The mean time to perform the TUG test was 12.4 ± 4.6 s. This test could not be performed in eight patients. Less than 10% of patients were classified as probable sarcopenic (*n* = 39; 7.1%), while 15.1% (*n* = 83) were diagnosed as sarcopenic and 2.5% (*n* = 14) as severe sarcopenic. More than one-third of the patients (*n* = 188; 34.2%) were diagnosed with malnutrition. The MSS was diagnosed in 55 patients (10%).

The incidence rate of in-hospital death was equal to 2.2% (*n* = 12), and the median LOS was 10.0 (5.0–18.0) days. Six months after discharge, it was possible to contact 520 patients (12 died in hospital and 18 did not answer the call); among them, 7.9% (*n* = 41) had died and 33.8% (*n* = 176) were readmitted.

### 3.2. Association between Sarcopenia and Clinical Outcomes

Table 1 presents the comparison of patients grouped according to the sarcopenia diagnosis. Non-sarcopenic patients were younger, presented higher BMI, HGS, and CC in comparison to the probable sarcopenic and sarcopenic ones. The median LOS was lower, as well as the incidence rate of death for non-sarcopenic patients, in-hospital, and at six months after discharge.

**Figure 1:**
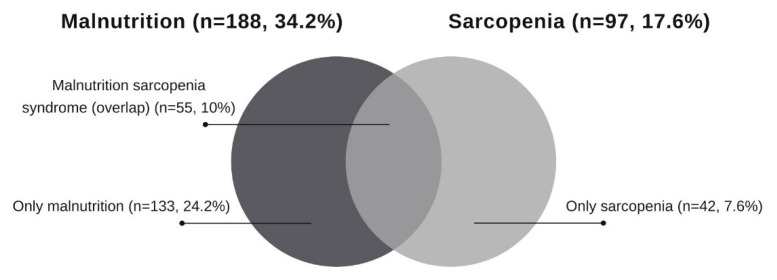
Overlap between malnutrition and sarcopenia in hospitalized patients (*n* = 550).

A higher frequency of malnutrition was diagnosed in sarcopenic patients, with more than half (*n* = 55; 56.7%) presenting it, while 43.3% (*n* = 42) of sarcopenic patients were diagnosed as well-nourished. In non-sarcopenic patients, 70.6% (*n* = 320) were well nourished, and 29.4% (*n* = 133) were diagnosed as malnourished. Figure 1 shows the diagram of percentages with malnutrition, sarcopenia, and MSS (Figure 1).

According to the multivariate analysis, the presence of sarcopenia increased the risk of in-hospital death by 3.95 times, and it was positively associated with higher odds (OR = 3.25) of mortality six months after discharge. On the other hand, it was not an independent predictor of prolonged LOS or hospital readmission in six months (Table 2).

Malnourished patients also presented a higher risk of in-hospital death (HR = 3.62), higher odds of prolonged LOS (OR = 2.27), and of mortality six months after discharge (OR = 3.42), although it was not an independent predictor of six-month hospital readmission, as demonstrated in Table 2.

The MSS was associated with all outcomes assessed, increasing the risk of in-hospital death (it did not reach the statistical significance), the odds of prolonged hospital stay, and readmission six months after discharge, in a higher magnitude, compared to these conditions isolated (Table 3).

## 4. Discussion

As well established in the literature, our study confirmed that malnutrition is an independent predictor of in-hospital death, prolonged hospital stay, and mortality six months after discharge. In addition, we demonstrated that sarcopenic patients present a higher risk of death (in-hospital and six months after discharge). The malnutrition–sarcopenia syndrome was observed in 10% of the sample and was a predictor of a higher risk of worse outcomes compared to these conditions isolated.

While 24% of patients presented sarcopenia and 34% were diagnosed with malnutrition, MSS was identified in 10% of our sample. The pooled data of seven studies, included in a systematic review conducted with older hospitalized patients, showed an overlapping prevalence of malnutrition–sarcopenia and sarcopenia-risk of malnutrition equal to 20.3% and 21.3%, respectively [19]. The higher prevalence could be justified by the older adults included in the primary studies pooled in the meta-analysis, and different tools used for malnutrition diagnosis in them (mini-nutritional assessment, nutritional risk screening, and malnutrition universal screening tool). It is well known that sarcopenia and malnutrition are more common in older patients [4,8].

In previous studies, sarcopenia has been associated with prolonged LOS. A meta-analysis of 15 studies demonstrated a mean difference of 4.54 days (95% CI 2.49–6.59 days) in sarcopenic gastrointestinal oncological patients undergoing surgery, compared to non-sarcopenic ones [15]. A positive association of sarcopenia and prolonged LOS was also demonstrated in hemiplegic stroke patients (>60 years) in a study that applied the Asian Consensus for sarcopenia diagnosis [*n* = 66; OR 1.19 (95% CI 1.02–1.38)] [28], and in hospitalized older adults with heart failure [*n* = 64,476; OR 1.40 (95% CI 1.37–1.43)] [29]. In the current study, sarcopenia was not an independent predictor of prolonged hospital stay, and the short hospital stay of patients in our sample could justify it. In addition, different definitions, tools, and cutoff points to identify sarcopenia in the studies, and features of the sample (e.g., age, ethnicity), make it difficult to compare the results regarding the prognostic value of sarcopenia in predicting prolonged LOS [30]. On the other hand, the MSS increased by 2.73 times the odds of prolonged LOS in our study, while malnutrition increased it by 2.23 times. Until this moment, we have not found other studies investigating the magnitude of association between MSS and prolonged hospital stay to compare our results.

In-hospital mortality is a public health and patient safety concern, also used as an indicator of hospital quality care [31]. Sarcopenia was a significant indicator for in-hospital mortality in our study (HR = 3.95). Following our results, a multicenter study evaluating older adults admitted to acute care wards (*n* = 770) showed that sarcopenia was significantly associated with in-hospital mortality (HR: 3.45; 95% CI 1.35–8.86) [32]. The MSS has increased the magnitude of association with in-hospital death (HR = 4.95) but did not reach statistically significant *p* Value. A higher risk of death in patients with MSS was demonstrated by another study with 453 older hospitalized patients [20]. According to the authors, this condition may serve as a prognostic factor in the management of hospitalized older patients.

Patients with sarcopenia and other nutritional syndromes can experience long-term complications that may lead to hospital readmission or even death. Xi et al. [33], evaluating patients (*n* = 485) after abdominal trauma, found that sarcopenia was an independent predictor of 90-day readmission (OR: 5.34; 95% CI 2.52–11.3). The afforded mentioned study had a shorter follow-up period and only evaluated sarcopenic patients, with a higher number of patients with this condition (*n* = 120, 24.7%) compared to our study. This might explain the reason we found no association between sarcopenia and hospital readmission in the six-month follow-up in our study. Malnutrition was also not associated with increased odds for hospital readmission. Ruiz et al. [34] did not find an association between hospital readmission and malnutrition. The authors suggest that several factors (low hospital readmission, health care system, a small number of patients with malnutrition) explain these results. However, MSS increased the odds for hospital readmission more than seven times. Until now, we did not identify other studies assessing the association between this syndrome and hospital readmission. Future studies will confirm if the MSS is more unfavorable for hospitalized patients than these conditions isolated, regarding hospital readmission.

Malnutrition, sarcopenia, and MSS were independent predictors of mortality six months after discharge. In a large cohort study involving geriatric inpatients (*n* = 1406), sarcopenia was associated with 3-month and 1-year mortality in males (HR 3.72; 95% CI 2.06–6.72; HR 1.92; 95% CI 1.34–2.77) and females (HR 2.95; 95% CI 1.56–5.60; HR 2.93; 95% CI 1.89–4.54) [35]. Studies involving patients with different types of cancer, pooled in a systematic review with meta-analysis, showed an overall HR on cancer mortality of 1.69 (95% CI 1.56–1.83) for patients with sarcopenia [36]. In a bi-centered prospective cohort study conducted with older individuals, MSS, sarcopenia, sarcopenia with malnutrition risk, and malnutrition groups were independently associated with all-cause mortality at two years. The MSS group had the highest HR (19.8) [12]. These results, combined with ours, confirm the hypothesis that the prognosis of patients with the syndrome is worse than in those with only one of these conditions of nutrition status impairment.

The present study has some limitations that should be considered. The single-center recruitment and some inclusion criteria (patients with the possibility to communicate and walk, able to have HGS measured, without leg edema) may have influenced our sampling, not allowing the extrapolation of the results to all hospitalized patients. However, these criteria are indispensable for the application of tools for the diagnosis of malnutrition and sarcopenia. Although the EWGSOP2 recommends using a screening tool for sarcopenia before the confirmation, the screening tools available have not been validated for the hospitalized population. Therefore, we evaluated the presence of probable sarcopenia and sarcopenia in all patients and grouped them in a more conservative approach, since we have already demonstrated in a previous study that reduced HGS is an independent predictor of worse outcomes in hospitalized patients [37]. In addition, even though the proposed diagnosis for primary sarcopenia focuses on elderly patients, we included both adults and the elderly in our analysis, as other studies cited previously. Together, the results suggest that the proposed diagnosis criteria are also sensible to predict worse clinical outcomes in adults presenting secondary sarcopenia.

There are limited data available in the literature on the relationship between MSS and clinical outcomes other than mortality. Our study showed that the co-existence of both conditions increases the prognostic value for adverse clinical outcomes in hospitalized patients, as prolonged LOS, readmission, and mortality six months after discharge. These findings serve to draw attention to the conundrum of characterizing the MSS [20]. Both must be addressed based on their diagnostic criteria, as they can coexist, and produce an additive effect on morbidity and mortality. The new diagnostic criteria from EWGSOP2, focusing on HGS to assess sarcopenia, is more accessible and practical [38]. In addition, in the current study, we used reduced CC to identify reduced muscle mass, and it is also a more accessible alternative for clinical practice. Its prognostic value has already been demonstrated in hospitalized patients and its agreement with reference methods for muscle mass assessment [39,40]. We used SGA to diagnose malnutrition since it is considered the reference method for hospitalized patients [26]. However, alternative methods for malnutrition have been proposed [4], and we have already demonstrated their satisfactory concurrent validity—AND-ASPEN Consensus and GLIM criteria—with SGA [22]. Therefore, combining them with sarcopenia diagnosis to identify MSS should be the focus of future studies. Added to this, the adjusted cutoff point of CC for BMI recently proposed for the American population [41] should be confirmed in other populations, especially in hospitalized patients, to be considered in sarcopenia diagnosis in the future. In addition, future studies could explore the association between metabolic diseases and decreased activity in hospitalized patients, since body composition and muscle strength may depend on the underlying disease. Our data preclude a stratified analysis by different groups of metabolic diseases (i.e., lung diseases, endocrine diseases, cardiovascular diseases) due to our sample size (insufficient for this), and how our data were collected and stored. However, the higher values of age-adjusted CCI observed in sarcopenic patients suggest a possible association between burden-disease and reduced activity of hospitalized patients.

## 5. Conclusions

Hospitalized patients with sarcopenia (isolated or co-existing with malnutrition), at admission, have suffered an increased risk of adverse outcomes. The malnutrition–sarcopenia syndrome was present in 10% of the sample and associated with a higher chance of prolonged hospital stay and hospital readmission six months after discharge, in comparison to these conditions isolated. These findings reinforce the importance of early identification of sarcopenia and its overlap with malnutrition, and the need for clinical guidelines for its management in hospital settings, to target patient care and improve their prognosis.

## Figures and Tables

**Table 1:** General and clinical outcomes comparison of patients grouped according to sarcopenia diagnosis.

	Non-Sarcopenic Patients (*n* = 453)	Sarcopenic Patients (*n* = 97)	*p* Value
Age (years)	54.7 ± 14.6	58.0 ± 15.9	**0.049** ^1^
Males	243 (53.9%)	48 (49.5%)	0.501 ^2^
White ethnicity	352 (77.7%)	73 (75.3%)	0.698 ^2^
Cancer diagnosis	249 (55.0%)	46 (47.4%)	0.215 ^2^
Surgical procedure	328 (72.4%)	63 (64.9%)	0.178 ^2^
BMI (kg/m^2^)	28.6 ± 5.2	22.1 ± 3.4	**<0.001** ^1^
Malnutrition (SGA B or C)	133 (29.4%)	55 (56.7%)	**<0.001** ^2^
HGS (kg)	29.2 ± 10.5	22.2 ± 8.0	**<0.001** ^1^
CC (cm)	37.7 ± 3.7	31.0 ± 2.3	**<0.001** ^1^
TUG (seconds)	12.0 ± 4.7	13.9 ± 3.6	0.229 ^1^
CCI age adjusted	4.0 (2.0–6.0)	4.0 (2.5–7.5)	0.066 ^3^
LOS (days)	10.0 (4.5–17.0)	10.0 (4.5–24.5)	**0.033** ^3^
In-hospital death	4 (0.9%)	8 (8.2%)	**<0.001** ^4^
Hospital readmission *	145 (33.2%)	31 (37.3%)	0.542 ^2^
Six-month death *	26 (5.9%)	15 (18.1%)	**<0.001** ^2^

Abbreviations: BMI, body mass index; SGA, subjective global assessment; HGS, handgrip strength; CC, calf circumference; TUG, timed up-and-go test; CCI, Charlson comorbidity index; LOS, length of hospital stay. ^1^ Student *t*-test; ^2^ chi-square test; ^3^ Mann–Whitney test; ^4^ exact Fisher test. * *n* = 520. Data are presented as mean ± standard deviation or median (P25–P75) or *n* (%); Number in bold indicate statistically significant results.

**Table 2:** Association between probable sarcopenia or sarcopenia and clinical outcomes: multivariate analysis.

Independent Variable	Sarcopenia		Malnutrition	
Dependent Variable	OR ^1^/HR ^2^ (95% CI)	*p* Value	OR ^1^/HR ^2^ (95% CI)	*p* Value
Prolonged LOS (>10 days) ^1^	1.23 (0.77–1.97)	0.382	2.27 (1.57–3.28)	**<0.001**
In-hospital death ^2^	3.95 (1.11–13.91)	**0.034**	3.62 (1.09–11.95)	**0.035**
Readmission within six months ^1^	1.09 (0.66–1.80)	0.741	1.27 (0.87–1.84)	0.224
Death within six months ^1^	3.25 (1.56–6.62)	**0.001**	3.42 (1.82–6.45)	**<0.001**

Abbreviations: CCI, Charlson comorbidity index; CI, confidence interval; HR, hazard ratio; LOS, length of hospital stay; OR, odds ratio. ^1^ Logistic regression. ^2^ Cox regression. Multivariate analysis adjusted for CCI and surgical procedure during hospital stay. Number in bold indicate statistically significant results.

**Table 3:** Association of sarcopenia or/and malnutrition with clinical outcomes: multivariate analysis.

**Predictors**	**Prolonged LOS (>10 Days)**		**In-Hospital Death**	
	**OR (95% CI) ^1^**	***p* Value**	**HR (95% CI) ^2^**	***p* Value**
Without malnutrition or sarcopenia	Ref.	-	Ref.	-
With malnutrition or sarcopenia	1.70 (1.15–2.51)	**0.008**	2.71 (0.41–17.83)	0.300
Malnutrition–sarcopenia syndrome	2.73 (1.42–5.25)	**0.003**	4.95 (0.98–25.17)	0.054
	**Readmission within Six Months**		**Death within Six Months**	
	**OR (95% CI) ^1^**	***p* Value**	**OR (95% CI) ^1^**	***p* Value**
Without malnutrition or sarcopenia	Ref.	-	Ref.	-
With malnutrition or sarcopenia	2.69 (1.24–5.86)	**<0.001**	1.64 (0.84–3.20)	0.147
Malnutrition–sarcopenia syndrome	7.64 (3.06–19.06)	**<0.001**	1.15 (1.08–1.21)	**<0.001**

Abbreviations: CCI, Charlson comorbidity index; CI, confidence interval; HR, hazard ratio; LOS, length of hospital stay; OR, odds ratio. ^1^ Logistic regression. ^2^ Cox regression. Multivariate analysis adjusted for CCI and surgical procedure during hospital stay. Number in bold indicate statistically significant results.

## Data Availability

Data available on request due to ethical and privacy restrictions. The data presented in this study are available on request from the corresponding author. The data provided by the volunteers are not publicly available due to privacy/ethical restrictions.
